# Relationship between insulin and Netrin-1/DCC guidance cue pathway regulation in the prefrontal cortex of rodents exposed to prenatal dietary restriction

**DOI:** 10.1017/S204017442300017X

**Published:** 2023-07-11

**Authors:** Aashita Batra, Santiago Cuesta, Marcio Bonesso Alves, Jose Maria Restrepo, Michel Giroux, Daniela Pereira Laureano, Amanda Brondani Mucellini Lovato, Patrícia Maidana Miguel, Tania Diniz Machado, Roberta Dalle Molle, Cecilia Flores, Patricia Pelufo Silveira

**Affiliations:** 1 Integrated Program in Neuroscience, McGill University, Montreal, QC, Canada;; 2 Ludmer Centre for Neuroinformatics and Mental Health, McGill University, Montreal, QC, Canada;; 3 Douglas Mental Health University Institute, McGill University, Montreal, QC, Canada;; 4 Department of Cell Biology and Neuroscience, Rutgers University, New Brunswick, NJ, USA;; 5 Department of Psychiatry, McGill University, Montreal, QC, Canada;; 6 Programa de Pós-Graduação em Saúde da Criança e do Adolescente, Faculdade de Medicina, Universidade Federal do Rio Grande do Sul, Porto Alegre, Brazil;; 7 Programa de Pós-Graduação em Neurociências, Instituto de Ciências Básicas da Saúde, Universidade Federal do Rio Grande do Sul, Porto Alegre, Brazil;; 8 Programa de Pós-Graduação em Psiquiatria e Ciências do Comportamento, Faculdade de Medicina, Universidade Federal do Rio Grande do Sul, Porto Alegre, Brazil; 9 Department of Neurology and Neurosurgery, McGill University, Montreal, QC, Canada

**Keywords:** Prenatal adversity, Intrauterine growth restriction, Food restriction, Insulin, Axonal guidance cues, Mesocorticolimbic dopamine pathway

## Abstract

Fetal restriction (FR) alters insulin sensitivity, but it is unknown how the metabolic profile associated with restriction affects development of the dopamine (DA) system and DA-related behaviors. The Netrin-1/DCC guidance cue system participates in maturation of the mesocorticolimbic DA circuitry. Therefore, our objective was to identify if FR modifies Netrin-1/DCC receptor protein expression in the prefrontal cortex (PFC) at birth and mRNA in adulthood in rodent males. We used cultured HEK293 cells to assess if levels of miR-218, microRNA regulator of DCC, are sensitive to insulin. To assess this, pregnant dams were subjected to a 50% FR diet from gestational day 10 until birth. Medial PFC (mPFC) DCC/Netrin-1 protein expression was measured at P0 at baseline and *Dcc*/*Netrin-*1 mRNA levels were quantified in adults 15 min after a saline/insulin injection. miR-218 levels in HEK-293 cells were measured in response to insulin exposure. At P0, Netrin-1 levels are downregulated in FR animals in comparison to controls. In adult rodents, insulin administration results in an increase in *Dcc* mRNA levels in control but not FR rats. In HEK293 cells, there is a positive correlation between insulin concentration and miR-218 levels. Since miR-218 is a *Dcc* gene expression regulator and our in vitro results show that insulin regulates miR-218 levels, we suggest that FR-induced changes in insulin sensitivity could be affecting *Dcc* expression via miR-218, impacting DA system maturation and organization. As fetal adversity is linked to nonadaptive behaviors later in life, this may contribute to early identification of vulnerability to chronic diseases associated with fetal adversity.

## Introduction

Reduced growth in fetal life has been strongly linked to the development of impaired glucose tolerance and type II diabetes,^1^ mainly due to altered growth and development of the pancreas.^2^ Insulin secretion relative to insulin sensitivity is highly impaired in these individuals throughout their lifetime as a consequence of poor growth before birth.^3–8^ Many models have shown that reduced growth due to poor nutrition increases the risk for adult chronic diseases. One of the most important models being that of caloric restriction^1,9,10^ because it re-capitulates many features seen in intrauterine growth restriction (IUGR) cases in humans such as catch-up growth,^11^ hyperinsulinemia,^12^ type II diabetes,^13^ and altered behaviors such as increased preference for palatable foods^9^ and impulsivity.^14^ Impulsivity includes two dimensions: action and choice.^15^ Impulsive action refers to the inability in the inhibition of response such as responding without thinking^16^ while impulsive choices involve a decision-making process and are expressed by the tendency to prefer immediate gains over long-term ones.^17,18^ Both action and choice impulsivity involve proper function of the mesocorticolimbic dopamine (DA) system.^19^

It is yet to be fully established how IUGR-induced changes in insulin alter impulsivity and whether regulators of mesocorticolimbic DA development are involved. Tyrosine hydroxylase (TH) is the rate-limiting enzyme which converts L-tyrosine to L-3,4-dihydroxyphenylalanine, which ultimately turns into DA.^20^ In response to sweet food intake, adult fetal growth restricted (FR) animals, meaning offspring of female rodents that were subjected to caloric restriction during pregnancy, show an increase in the expression of TH in the prefrontal cortex (PFC) in comparison to controls (referred to as AdLib in the manuscript as the dams were fed ad libitum diet).^9,21^ Since DA in the PFC is involved in attention and impulsivity-related behaviors in rats, increased TH expression in FR animals could be associated to the animals’ reward-associated value of sweet food, as evident by Alves et al. where the mean change in DA signaling in PFC was measured in controls and FR animals in response to standard chow and Froot Loops^®^ consumption.^22^ They observed that Froot Loops^®^ consumption caused a blunted increase in DA concentration in FR animals when compared to AdLib animals.^22^ Another study showed that FR animals exhibited higher peripheral insulin levels than AdLib animals^23^ suggesting that IUGR modifies the modulation of mesolimbic DA neurons by insulin beyond the well-known alteration in peripheral glucose metabolism.^22^ In controls, the PFC DA levels in response to Froot Loops^®^ consumption were greater than the levels in response to standard chow, while FR animals did not show a difference in DA levels in response to chow and Froot Loops^®^. The weakened response to sweet food seen in the PFC of IUGR animals may result from decreased activation of striatal areas, leading to a lower valuation of the reward magnitude and therefore, favoring impulsive choices for immediate small rewards.^21^ Within the PFC, there seems to be a positive correlation between impulsive choice and expression of D1 receptors^19^ since micro-infusion of D1 receptor antagonist in the area increased impulsive choice. Altered expression of D2 receptors, which are found to be increased in the PFC of IUGR animals at 160 d of life, is also linked with impulsive choice.^24^ Together, these findings indicate that fetal adversity alters brain sensitivity to insulin and modulates behavioral responses to palatable foods.^22,23^ Additionally, in rodents exposed to IUGR, there is systemic hyperglycemia/hyperinsulinemia^25^ and alterations in the expression of genes or proteins associated with DA signaling in the PFC, including TH, pTH, D1, and D2.^9,21,23,26^ These metabolic and neurochemical effects are accompanied by changes in cognitive flexibility, sensitivity to reward, and poor inhibitory control.^9,21,22,26,27^ The behavioral and metabolic phenotype observed in animals exposed to FR recapitulates the profile detected in humans born after exposure to prenatal adversity^28–48^ with striking accuracy. In humans, differences in DA function associate with the increased palatable food consumption in IUGR individuals.^49^ Furthermore, Silveira et al. discovered, in two independent cohorts, that catch-up growth, an insulin-dependent process, influences decision-making and inhibitory control specifically in IUGR children tested in tasks that did not involve the choice for food rewards.^14^ Given the involvement of the mesocorticolimbic DA pathway in impulsive behaviors, we are interested in investigating the molecules involved in the maturation of this pathway and characterize the effect of early life adversity in this developmental process by using the FR animal model.

The guidance cue Netrin-1 participates in the developmental organization of neural networks by either attracting or repelling extending axons and dendrites and is highly expressed in terminal fields of mesocorticolimbic DA neurons including the PFC.^50^ Responses to Netrin-1 can be modulated by regulating the availability of DCC (deleted in colorectal cancer) and UNC5 (uncoordinated) receptors. DCC receptors in DA axons promote target recognition events in the NAcc, preventing them from continuing to grow to the PFC,^51–55^ and thereby organizing PFC local circuits^50,56,57^ and inhibitory control in adulthood.^56,57^ DCC receptors are also expressed by PFC local neurons across the lifespan and are involved in susceptibility/resilience to chronic social defeat stress.^58^ Changes in DCC expression in DA and cortical neurons induced by exposure to drugs of abuse or to stress, respectively, have been shown to be mediated by the microRNA-218 (miR-218).^58,59^ miR-218 can be detected in blood with circulating levels matching its expression in brain.^60,61^ Both chronic caloric restriction and chronic high fat diet exposure in adulthood increase the expression of miR-218 in the hypothalamus.^62^ However, the possible persistent effect of dietary manipulations during fetal development, as well as the effect of differences in insulin sensitivity on the expression of miR-218 and its guidance cue receptor targets in the PFC remain unknown.

Based on these findings, we aimed to investigate if exposure to prenatal dietary restriction alters guidance cue systems known to be intimately involved in DA, PFC, cognitive development, and in vulnerability to chronic stress. Using the FR model of IUGR in male rats, we measured changes in the Netrin-1/DCC guidance cue pathway in the PFC at birth and in adulthood and assessed if alterations in insulin sensitivity and microRNA levels are involved in the process.

## Methods

Primiparous Sprague Dawley rats of approximately 70–80 d were maintained in a controlled environment: standard dark/light cycle of 12 h each, ambient temperature of the colony housing and testing rooms was 22 ± 1°C, relative humidity level was approximately 20–50%, cage cleaning once a week, and food and water provided *ad libitum*. Estrous cycle was determined daily by vaginal smearing and females were time mated. Gestation was confirmed at day 1 by visualizing the presence of sperm cells on the vaginal smear. On gestational day 10, dams were randomly allocated into one of the following dietary groups: control group (AdLib), which received an *ad libitum* diet of standard laboratory chow (3.1 kcal/g, 24% protein, 18% fat, 58% carbohydrate; Teklad Diets^®^), or a 50% food restricted group (FR), based on the IUGR model described by Desai et al.,^63^ which received 50% of the *ad libitum*-fed dams’ intake (determined by daily quantification of normal intake in a separate cohort of pregnant Sprague Dawley rats). The food restriction occurred from day 10 of pregnancy until the pups were born. The average calories consumption from day 10 to day 22 of pregnancy was 103.4 ± 9.3 kcal/d in AdLib group and 51.7 ± 4.6 kcal/d in FR group. Belkacemi et al.^64^ showed that this IUGR model increases FR dams’ plasma corticosterone levels compared to AdLib dams, mimicking a stressful environment.

Within 24 h of birth, pups from each litter were individually weighed and standardized to a maximum of eight pups per litter, with four females and four males. Cross-fostering was performed, and all litters were adopted by AdLib dams. Only male offspring were used in this study and only one animal per litter was used in each experiment. Tissues were collected in two separate age groups. The first group’s tissue was collected on the day the animals were born. The second group’s pups were weaned on postnatal day (PND) 21, separated into groups of two same-sex (same litter/group) individuals per cage, and kept in a controlled environment as previously described. Except for the cage cleaning once a week, animals were left undisturbed from PND 21 until tissue collection which occurred at approximately PND 100.

### Tissue collection

Tissue collection for protein level analysis took place at two different ages: PND 0 and PND 100. For the PND 0 group, the animals were decapitated, the brains were quickly removed and a series of 0.5 mm brain punches from the PFC were dissected from brain coronal sections corresponding to plates 7–9 of the Brain Rat Atlas.^65^ Tissue was stored at −80°C until further analysis. For the PND 100 group, the animals received either a peripheral saline injection or insulin injection. Fifteen minutes after the injection, the animals were decapitated, the brains were quickly removed, the PFC was dissected through 1.0 mm thick coronal slices with the aid of an atlas,^66^ and the tissue was stored at −80°C until further analysis.

### Western blot

Total protein fractions were isolated using the mirVana^™^ PARIS RNA and Native Protein Purification Kit Protocol (Cat#AM1556, Thermo Scientific, Toronto, ON, Canada). Briefly, protein samples (20 μg) were separated on a 10% SDS-PAGE and transferred to a PVDF membrane that was incubated overnight at 4°C with antibodies against DCC (1:1000, Cat#554223, BD Pharmingen), Netrin-1 (1:1000, cat# ab126729, Abcam), and alpha-tubulin (1:2000, Cat #2144S, Cell Signaling). We obtained the optical density for each Netrin-1, DCC, and tubulin band, and the fold change was calculated by normalizing to tubulin’s optical density.

### Tissue RNA extraction and quantitative real-time PCR

Total RNA and microRNA fraction were isolated from the frozen tissue using the Norgen Biotek Corp. RNA/DNA/Protein Purification Plus Kit (Cat#47700, Norgen Biotek Corp, Thorold, ON, Canada) as per manufacture’s instruction. All RNA samples were determined to have 260/280 and 260/230 values ≥1.8, using the Nanodrop 1000 system (Thermo Scientific, Toronto, ON, Canada). Reverse transcription for *Dcc*, *Netrin-1* (*Ntn-1)*, and *Glyceraldehyde-3-phosphatedehydrogenase* (GAPDH) mRNA were performed using a High-Capacity cDNA Reverse Transcription Kit (Applied Biosystems, Foster City, CA) according to manufacturer’s instructions. Real-time PCR, using TaqMan assay (Applied Biosystems, Foster City, CA) was carried out with an Applied Biosystems 7900HT RT PCR system. Data for *Dcc* and *Ntn-1* mRNA expression were analyzed by using the Relative Quantification standard curve method and *Gapdh* was used as reference gene. In all cases, the real-time PCR was run in technical triplicates.

### HEK293 in vitro analysis

HEK293 cells were cultured in 12-well plates with DMEM medium with 10% fetal bovine serum (FBS). Once the cells reached 80% of confluency, they were starved in serum-free medium for 16–18 h. After that period two different treatments were applied: (1) exposure to different concentrations of insulin (50, 100 or 200 nM) or basal medium to measure total micro-RNA levels after 6 h and (2) exposure to 100 mM of insulin or basal medium to measure total micro-RNA levels at different time points (10, 30, 60 or 360 min). In both cases, a control, undisturbed cell culture was kept (UNT). Cell cultures were harvested by trypsinization (0.125 trypsin/1% EDTA in PBS) and homogenized using the miRNeasy Mini Kit (Qiagen, Toronto, ON, Canada). Total RNA and microRNA fraction were isolated according to the manufacturer’s instructions. All RNA samples were determined to have 260/280 and 260/230 values ≥1.8, using the Nanodrop 1000 system (Thermo Fisher Scientific, Toronto, ON, Canada). Reverse transcription for miR-218 was performed using the TaqMan MicroRNA Reverse Transcription Kit and TaqMan probes (Applied Biosystems, QC, Canada). Real-time PCR was run in technical triplicates with an Applied Biosystems QuantStudio RT PCR system (Applied Biosystems, QC, Canada). The small nucleolar RNA (snoRNA) RNU6B was used as an endogenous control of miRNA measures. Expression levels of miR-218 were calculated using the Relative Quantitation standard curve method and normalized by RNU6B. The expression of miR-218 was relativized to UNT group and presented as a fold change.

### Statistical analysis

Student’s *t*-test was used for the Western Blot analysis for PND 0 animal results. All variables were expressed as mean ± standard error of the mean (SEM). One-way ANOVA was used to analyze the Western Blot analysis for PND 100 animal results and the in vitro results from HEK293 cultures followed by Tukey post hoc test when appropriate. Results were considered significant when *p* ≤ 0.05. Data were analyzed using the RStudio software.^67^

## Results

### PND 0 measures

**Figure 1.**
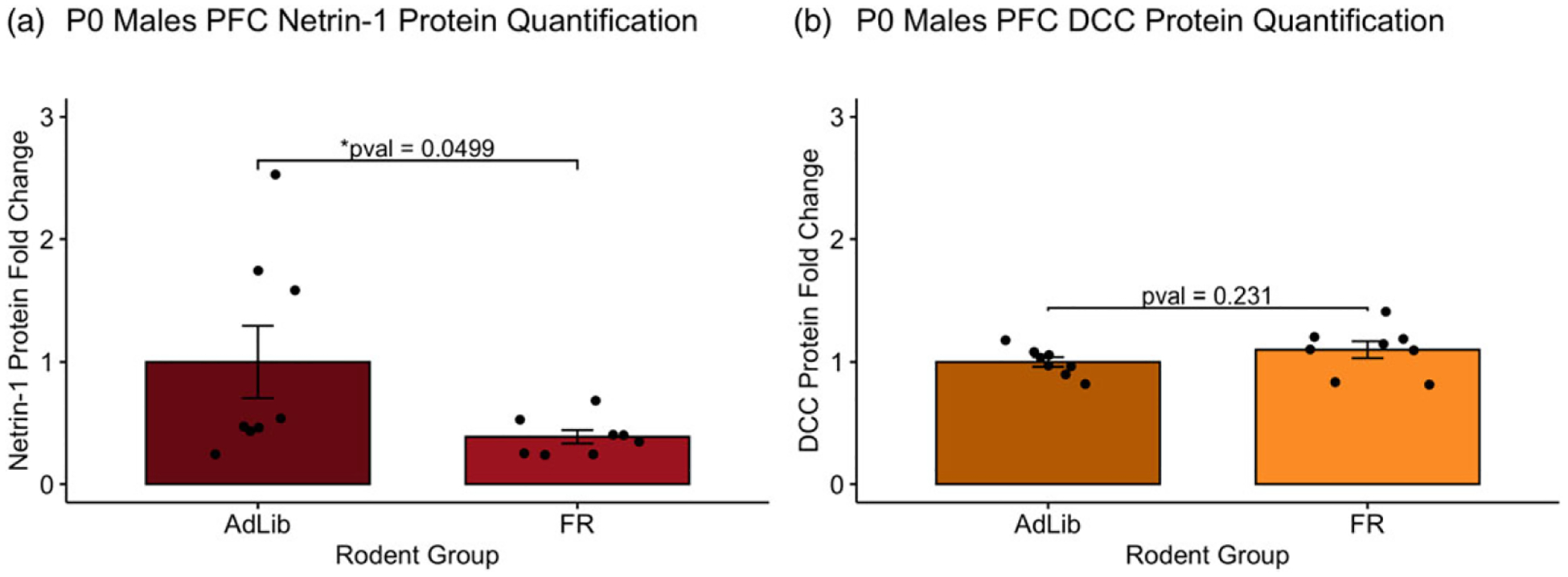
PFC Netrin-1 and DCC expression in PND0 males. Levels of Netrin-1 and DCC receptors in the PFC were measured in AdLib and FR animals of age PND 0. (*a*) Netrin-1 protein levels were found to be downregulated in the PFC of FR animals when compared to AdLib animals (*W*(8) = 51; *p* = 0.0499). (*b*) DCC protein expression was not significantly different in the PFC of FR animals when compared to AdLib animals (*t*(14) = −1.25; *p* = 0.231).

Protein levels of Netrin-1 and DCC receptors in the PFC were measured in AdLib and FR animals of age PND 0 (AdLib weight = 7.55 ± 0.21, FR weight = 6.84 ± 0.11, Student’s *t*-test, *n* = 8/group, *t*(14) = 3.039; *p* = 0.009) as shown in Fig. 1. Netrin-1 protein levels were found to be downregulated in the PFC of FR animals when compared to AdLib animals (*W*(8) = 51; *p* = 0.0499). DCC protein expression was not significantly different in the PFC of FR animals when compared to AdLib animals (*t*(14) = −1.25; *p* = 0.231).

### PND 100 measures

**Figure 2.**
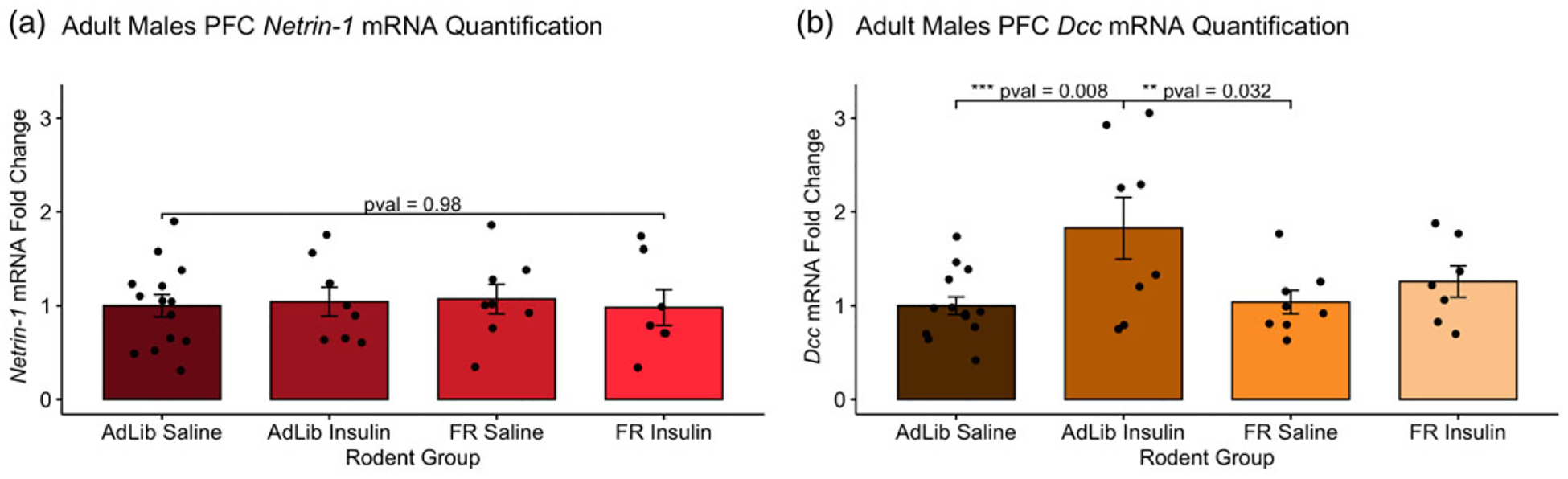
PFC *Netrin*-1 and *Dcc* mRNA expression in PND 100 males. Levels of *Netrin-1* and *Dcc* in the PFC were measured 15 min after a peripheral saline or insulin injection in AdLib and FR animals. (*a*) There were no significant differences in Netrin-1 mRNA levels across the four groups: AdLib Saline, AdLib Insulin, FR Saline, and FR Insulin (*F*(3,33) = 0.066, *p* = 0.98). (b) There was a significant difference in Dcc mRNA levels over groups (*F*(3,33) = 4.41, *p* = 0.0103). There is a significant upregulation of Dcc mRNA in AdLib animals treated with an insulin injection when comparing to AdLib animals administered a saline injection (*p* = 0.008) and a significant downregulation in *Dcc* mRNA levels in FR animals treated with the saline injection when compared to the AdLib animals given the insulin injection (*p* = 0.032).

Levels of *Ntn-1* and *Dcc* mRNA in the PFC were measured 15 min after an intraperitoneal saline or insulin injection after a 4 h fast (1 IU/kg) in AdLib and FR animals of age PND 100 as shown in Fig. 2. There were no significant differences in *Ntn-1* mRNA levels between the four groups: AdLib Saline, AdLib Insulin, FR Saline, and FR Insulin (*F*(3,33) = 0.066, *p* = 0.977). There was a significant difference in *Dcc* levels across the four groups (*F*(3,33) = 4.407, *p* = 0.0103). A Tukey post hoc test revealed that there is a significant *upregulation* of *Dcc* mRNA in AdLib animals which received the insulin injection when comparing to AdLib animals which were administered a saline injection (*p* = 0.008). We also found a significant *downregulation* of *Dcc* mRNA levels in FR animals which received a saline injection when compared to the AdLib animals treated with insulin (*p* = 0.032).

### miR-218 measures in HEK293 cultures

**Figure 3.**
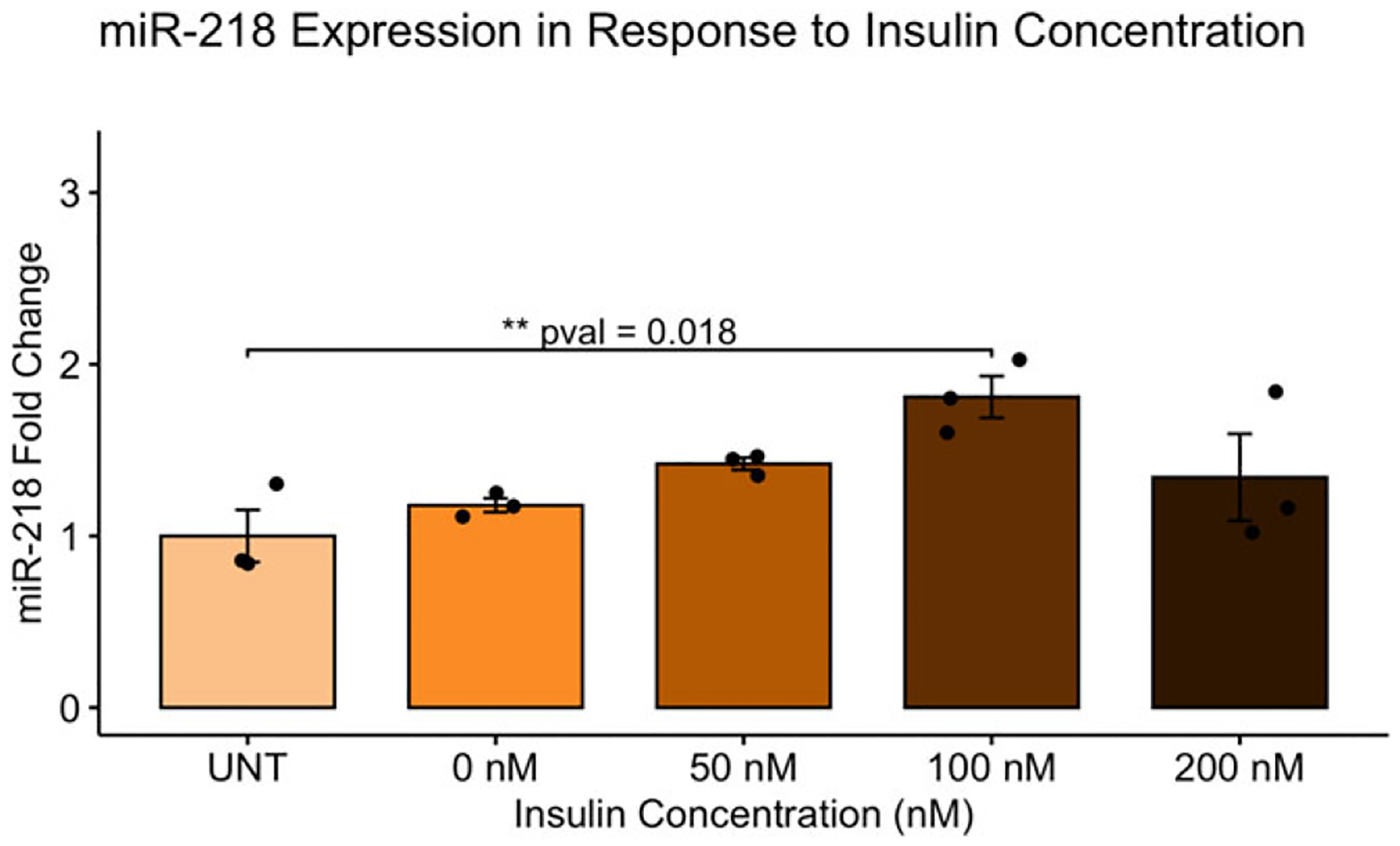
Insulin regulates miR-218 expression in a concentration-dependent manner. miR-218 expression was measured in response to four different insulin concentrations in addition to the control group (UNT): 0 nM, 50 nM, 100 nM, 200 nM. There was a significant difference in miR-218 expression across groups (*F*(4,10) = 4.394, *p* = 0.0262). miR-218 levels were upregulated at 100 nM of insulin when compared to the control group.

miR-218 expression was measured in response to four different insulin concentrations, as shown in Fig. 3, in addition to the control group (UNT): 0 nM, 50 nM, 100 nM, 200 nM. There was a significant difference in the fold change of miR-218 between the five groups (*F*(4,10) = 4.394, *p* = 0.0262). A Tukey post hoc test revealed that there is a significant *upregulation* in miR-218 in response to 100 nM of insulin (*p* = 0.018) when comparing to the control group.

**Figure 4.**
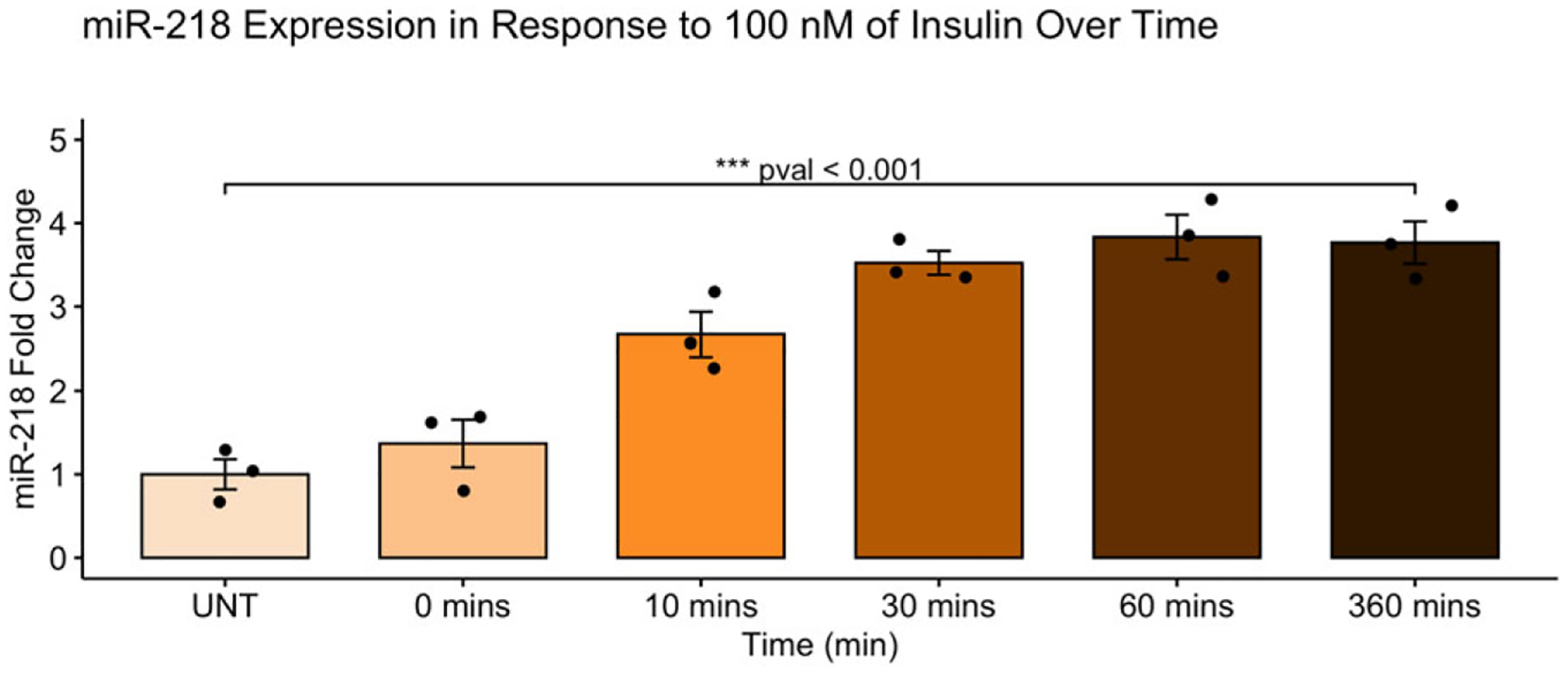
Effects of insulin signaling on miR-218 levels over time. Fold change in miR-218 were measured in response to 100 nM of insulin over a time curve at five different time points in addition to the control group (UNT): 0 min, 10 min, 30 min, 60 min, and 360 min. There was a significant difference in miR-218 expression across groups (*F*(5,12) = 27.26, *p* < 0.001). miR-218 levels were upregulated at 10 min, 30 min, 60 min, and 360 min in comparison to the control group.

miR-218 expression was measured over a time curve when exposed to 100 nM of insulin, as shown in Fig. 4, at five different time points in addition to the control group (UNT): 0 min, 10 min, 30 min, 60 min, and 360 min. There was a significant difference in miR-218 expression across the six groups (*F*(5,12) = 27.26, *p* < 0.001). A Tukey post hoc test revealed that there is a significant *upregulation* in miR-218 at 10 min (*p* = 0.004), 30 min (*p* < 0.001), 60 min (*p* < 0.001), and 360 min (*p* < 0.001) when comparing to the control group, *upregulation* at 10 min (*p* = 0.022), 30 min (*p* < 0.001), 60 min (*p* < 0.001), and 360 min (*p* < 0.001) when comparing to 0 min, and *upregulation* at 60 min (*p* = 0.043) when comparing to 10 min.

## Discussion

The food restriction model that we used in this study was based on the successful IUGR model described by Desai et al.^25^ The 50% food restriction protocol is quite stressful to the dams, increasing corticosterone levels at gestational day 20,^64^ and impacting offspring birth weight^21–23,68^ and brain weight at birth.^69^ This model was established as a useful approach able to mimic many of the adverse outcomes frequently observed in IUGR in humans, including decreased plasma leptin levels, increased food intake, obesity, increased percentage of body fat, insulin resistance, and catch-up growth.^70^ More recently, this model has been used to investigate alterations in feeding behavior that in some way influence food choices and consumption.^25^ In this context, interesting changes were found in attentional skills, in physical activity, in reward sensitivity (indicated by a decreased conditioning for palatable food in a place preference test), in hedonic responses to sweet food, and in impulsive behavior. Therefore, this model was best suited for our study where we investigated whether environmental insults early in life, modeled through prenatal dietary restriction, affect the Netrin-1/DCC guidance cue system which is known to be involved in DA and PFC maturation and function.^54,59,71,72^ We showed that Netrin-1 protein levels are downregulated in the PFC of FR animals in comparison to AdLib animals at age PND 0, indicating that prenatal dietary restriction affects developmental guidance cue systems as early as birth. In the matured cortex, at PND 100, we do not see changes in *Ntn-1* mRNA transcript expression at baseline (AdLib Saline group and FR Saline group) suggesting that the changes observed at birth do not persist into adulthood. FR does not alter baseline DCC protein expression in the PFC at birth or in mRNA transcript levels in adulthood. However, at PND 100, AdLib animals showed *Dcc* mRNA upregulation in response to an insulin challenge when compared to AdLib animals who received saline, but this response is absent in FR animals. This lack of regulation of *Dcc* by insulin in FR rodents is in line with our previous work showing that FR animals have different sensitivity to insulin^23^ and suggest that exposure to fetal growth restriction prevents insulin-induced regulation of *Dcc* expression in the PFC in adulthood. It was vital to test the effects of insulin on the guidance cues involved in the maturation of the DA pathway within the IUGR context because the IUGR model has been shown to affect insulin signaling and sensitivity within the brain. It is important to note that protein levels were measured in PND 0 animals while mRNA levels were measured in PND 100 animals but the positive correlation between mRNA and protein expression levels^73^ allows us to compare these transcripts between these ages.

The expression of the Netrin-1/DCC system in the mesocorticolimbic DA systems, including the PFC, decreases dramatically from early postnatal life to adulthood.^60,74,75^ This shift in expression levels coincides with the distinct role that Netrin-1 and DCC play across postnatal development – from axonal pathfinding and synaptic pruning/refinement in early postnatal life to synaptic plasticity in adulthood.^71,76^ It is likely that the early changes in Netrin-1 expression observed in FR animals impact the core organization of developing PFC neuronal networks, including DA axonal growth, while the altered insulin-response in DCC receptor expression in adulthood involve reorganization of matured local synaptic connectivity. In the PFC, Netrin-1 has been shown to be highly expressed by pyramidal and GABA interneurons, while DCC receptors are expressed by pyramidal neurons as well as non-DA axons.^50,75^ It remains to be determined whether changes in guidance cue expression induced by FR or by insulin administration in adulthood localize to specific PFC neuronal populations and if they are predominately occurring at the pre- or postsynaptic level.

miR-218 levels in the brain have been known to be affected by altered metabolism as shown by Sangiao-Alvarellos et al.^62^ where caloric restriction and high fat diet in adulthood resulted in an increase in miR-218 expression in the hypothalamus in comparison to the control group. Since miR-218 regulates *Dcc* mRNA and DCC protein expression,^59^ it could be involved in the effect of prenatal dietary restriction on this guidance cue system. To assess this hypothesis, we did in vitro analysis involving miR-218 in HEK-293 cells. We showed that there is a time-dependent and dose-dependent increase in miR-218 expression in HEK-293 cells. Whether and how insulin-induced regulation of miR-218 may be involved in the changes in DCC expression observed in the PFC in adulthood remains to be established. Indeed, previous studies have shown that miR-218 can stimulate the transcription of certain transcripts^77^ and cumulative evidence indicates that miRNAs can upregulate genes and protein expression in specific cell types and under particular conditions.^78^ Since miR-218 is a regulator of *Dcc* gene expression and our in vitro results show that insulin regulates miR-218 levels, we suggest that FR-induced changes in insulin sensitivity could be affecting *Dcc* expression via miR-218.

In conclusion, our work explores how insulin mediates the effects of prenatal dietary restriction on the Netrin-1/DCC guidance cue system which is known to be involved in PFC maturation. Previous animal work from our lab has shown that animals exposed to prenatal dietary restriction showed a pronounced aversion to delayed rewards in addition to an increase in PFC D2 levels.^22^ Our current findings may partially explain the mechanism involved in this relationship. This guidance cue pathway is affected by prenatal dietary restriction through changes in insulin, as shown by our results, which could ultimately reorganize pre- and postsynaptic networks and impact executive functions in adulthood. As the FR model parallels many of the alterations observed in IUGR in humans, the current findings have the potential to inform future studies to explore these mechanisms in human populations.
